# Identification of Putative Natriuretic Hormones Isolated from Human Urine

**DOI:** 10.3389/fendo.2015.00066

**Published:** 2015-05-20

**Authors:** Herbert J. Kramer

**Affiliations:** ^1^Center of Internal Medicine, Rheinische-Friedrich-Wilhelms-University, Bonn, Germany

**Keywords:** sodium transport, natriuretic hormone, human urine, endogenous inhibitors, epithelial sodium transport

## Abstract

This brief review describes some representative methodological approaches to the isolation of putative endogenous inhibitors of epithelial sodium transport – i.e., as ouabain-like factors (OLF) that inhibit the sodium transport enzyme Na-K-ATPase or inhibit the epithelial sodium channel (ENaC). Gel chromatography and reverse-phase (RP)-high performance liquid chromatography (HPLC) of lyophilized and reconstituted 24 h-urine from salt-loaded healthy humans led to two active fractions, a hydrophilic OLF-1 and a lipophilic OLF-2, whose mass (Ms)-spectroscopic data indicate a M_r_ of 391 (1, 2). Further identification was attempted by Ms-, infrared (IR)-, ultraviolet (UV)-, and ^1^H-NMR-spectroscopy. OLF-1 and OLF-2 may be closely related if not identical to (di)ascorbic acid or its salts such as vanadium (V)-V^v^-diascorbate with M_r_ 403 (3) and V^IV^-diascorbate. OLF-1 and V^v^-diascorbate are about 10-fold stronger inhibitors of Na-K-ATPase than OLF-2 and V^IV^-diascorbate, respectively. In conscious rats, i.v. infusion of OLF-1 and OLF-2 resulted in a strong natriuresis. In a similar study, Cain et al. (4) isolated a sodium transport inhibitor from the urine of uremic patients by gel chromatography and RP-HPLC. In uremic rats, a natriuretic response to the injection of the active material was found. Xanthurenic acid 8-O-β-d-glucoside (M_r_ 368) and xanthurenic acid 8-O-sulfate (M_r_ 284) were identified as endogenous inhibitors of sodium transport acting, e.g., by ENaC blockade. No definite relation to blood pressure, body fluid volume, or sodium balance has been reported for any of these above factors, and further studies to identify the natriuretic and/or ouabain-like compound(s) or hormone(s) will be needed.

## Introduction – Background

With this brief review, some methological aspects of the isolation of putative endogenous membrane transport inhibitor(s) and natriuretic factor(s) will be described, and the results compared with those of similar attempts by other groups of investigators.

In 1969 (5), and in more detail in 1974 (6) and 1977 (7), we demonstrated for the first time that acute extracellular fluid volume (ECFV)-expansion in rats may release a natriuretic factor or “hormone,” which was postulated to act through inhibition of the sodium pump. Thus, ECFV-expansion was accompanied by a decrease in Na-K-ATPase in the renal cortex and the appearance of an inhibitor of Na-K-ATPase in the serum of these rats, respectively. Using gel chromatography, this inhibitory activity was also detected in the post-salt fraction of serum from ECFV-expanded dogs and in the serum and urine of salt-loaded humans. Besides the *in vitro*-assay of the inhibitory activity, we also demonstrated the inhibitory effect of this serum fraction on epithelial sodium transport, i.e., on short-circuit current (SCC) and potential difference (PD) in the isolated frog skin (8). This fraction of serum or urine was also found to cause natriuresis in a rat bioassay. We concluded that a natriuretic factor emerges in the circulation with excessive salt load whose mechanism of action was to modulate the Na-K-ATPase enzyme in the vasculature as well as in the renal tubule.

Attempts to identify this humoral inhibitor(s), however, remained unsuccessful despite the use of extensive methodologic approaches. Besides an endogenous ouabain (9), several bufodienolides (10) have been identified using the methods described in this paper of which two compounds with M_r_ of around 400 daltons will be considered in the present mini-review, namely vanadium (V)-diascorbate(s) and derivatives of xanthurenic acid (4). The compounds were assumed to be present in the circulation, and therefore may be excreted in the urine.

## Ouabain-Like Factor(s) as Endogenous Sodium Transport Inhibitors

As source for isolation and identification of a putative natriuretic hormone and/or endogenous epithelial sodium transport enzyme inhibitor, we pooled large quantities (50–100 L) of urine from salt-loaded healthy humans, which was lyophilized to dryness and reconstituted with 0.01M acetic acid, and then subjected to gel chromatography using Sephadex G-25 and Sephadex G-10 columns.

### Ouabain-like factors and vanadium-diascorbic acid: Effects on Na-K-ATPase

To detect the serum and urine fractions with the active compound(s), we employed an *in vitro*-assay of Na-K-ATPase using a purified commercially available hog cerebral enzyme preparation. We also used this Na-K-ATPase membrane fraction as marker to follow-up activity with purification steps during the subsequent chromatographic steps. To detect the potential natriuretic activity, all fractions were screened for their natriuretic effect using a bioassay in conscious rats (11).

The transport enzyme Na-K-ATPase inhibitory and natriuretic activity(ies) eluted from the Sephadex G-25 column in a post-salt fraction. When this fraction was then subjected to gel chromatography on Sephadex G-10, a strongly active enzyme inhibitory material eluted in a late fraction (1). This late fraction also showed a significant natriuretic action (11). This enzyme inhibitory and natriuretic fraction was subjected to high performance liquid chromatography (HPLC), and subsequently to thin layer chromatography (TLC). Characterization of the active material was attempted by mass (M_r_)-, nuclear magnetic resonance (^1^H-NMR)-, infrared (IR)-spectroscopy (1), and ultraviolet (UV)-fluorescence/absorbance. The natriuretic activity was also studied by bioassay to identify the active compounds after gel filtration, reverse phase (RP)-HPLC, and amino acid analysis for its potential peptidic character (11).

Reverse-phase HPLC of this highly active late fraction from Sephadex G-10 resulted in two subfractions with significant Na-K-ATPase enzyme inhibition. They were named ouabain-like factors (OLF); one eluted in the water phase as the more polar hydrophilic OLF-1; the second eluted in a later phase at 20% acetonitrile as the more apolar lipophilic OLF-2. These fractions also produced a significant natriuresis (see below).

#### Analysis of Chemical Structure

Both compounds showed signals for hydroxyl and carboxyl groups as well as criteria for esters or lactones (a precursor of ascorbic acid in plants and animals is l-gulono-y-lactone, and 2,3-diketogulonic acid is an oxidation product of ascorbic acid). No signals for aromatic, aliphatic, heterocyclic, or steroid structures were found. Whereas the IR-spectrum of OLF-1 is different from that of OLF-2 (1), UV-, M_r_-, and ^1^H-NMR-criteria were similar and fluorescence of both compounds when separated by TLC required the presence of a dicarboxylic acid**-**like conformation; dicarboxylic acid [see also Ref. (11): Asp, Glu as carboxylic acids] is an organic compound containing two carboxyl functional groups (–COOH). IR- and ^1^H-NMR spectra of OLF-1 and OLF-2 suggest a chemical structure resembling a sugar or sugar derivative. However, sugars are not fluorescent as are the OLF recovered from TLC. Therefore, these data suggest the unknown compounds to be identical with ascorbic acid or its salts such as V^v^-diascorbate and V^lV^-diascorbate, respectively, with M_r_ 403 (3). The superscript roman numbers indicate the oxidative state of vanadium (V): V^lV^ oxide (V_2_O_5_), the most stable oxygen combination, and V^lV^ oxide (VO_2_) represent two of the four oxygen states of vanadium. V-diascorbates elute from the RP-HPLC column at similar elution times and acetonitrile gradients as the hydrophilic and lipophilic OLF-1 and OLF-2, respectively. V^IV^-diascorbate also showed the same UV-maximum as we found for OLF. Thus, ascorbic acid seems to be an important cornerstone of the structure of the yet unknown humoral ATPase inhibitor.

It is noteworthy that the water solubility of the individual ascorbic acid salts of metals varies remarkably, and it may be assumed that V^v^- and V^lV^ diascorbates with their different water solubility elute from the RP-HPLC column at similar elution times as the OLF-1 and OLF-2, respectively. V-diascorbates also show the same UV-maximum as the OLF and are strong candidates for the urinary hydrophilic OLF-1 and lipophilic OLF-2, respectively.

#### Effects on Enzyme Kinetics

These active subfractions, containing OLF-1 and OLF-2, were further purified by two-dimensional preparative TLC to single compounds, whose mass spectroscopic (MS) data suggested a M_r_ of around 400. Actually, OLF-2, which dose-dependently inhibited Na-K-ATPase, was found to have a M_r_ of 391 (1). With respect to the effects of OLF-1 and OLF-2 and of V^v^- and V^lV^-diascorbates on Na-K-ATPase enzyme activity and kinetics, *in vitro* studies showed that OLF-1 and OLF-2 inhibited the enzyme in its E2 configuration. In analogy to the polar OLF-1, which revealed an approximately 10-fold stronger enzyme inhibition (IC_50_ 1.5 × 10^−5^ M) than the apolar OLF-2 (IC_50_ 1.5 × 10^−4^ M), we found that V^v^-diascorbate (IC_50_ 2 × 10^−6^ M) is a significantly stronger inhibitor of Na-K-ATPase than V^IV^-diascorbate (IC_50_ of 9 × 10^−5^ M) (3, 5, 12). In this context, I should mention that we found previously that certain trace metals are strong inhibitors of this enzyme (13).

#### Renal and Vascular Mechanisms of Action of OLF

Regarding the potential mechanism of the physiological and pathological effects of OLF-1 and OLF-2 on vascular smooth muscle cells (VSMCs) and inner medullary collecting duct cells (IMCD cells), we found in an *in vitro*-assay that OLF-1 and OLF-2 enhanced VSMC contractility by increasing intracellular Ca^2+^ similar to the effect of ouabain (14, 15). Similar effects were found with OLF-1 and OLF-2 on intracellular Ca^2+^ in IMCD cells, suggesting inhibition of tubular Na-reabsorption and thus regulating renal excretion, i.e., to enhance Na-excretion (16).

### Ouabain-like factors and V-diascorbates: Natriuretic effects

For demonstration of the natriuretic activity, we used a bioassay in conscious rats (12). As mentioned above, in our assay system, the post-salt fraction IV from Sephadex G-25 was applied to Sephadex-G-10 and resulted in a late fraction, which was applied to RP-HPLC. When administered i.v., OLF-1 resulted in an immediate, eightfold rise in natriuresis from approximately 1 to 8 μEq/min/mg, whereas the apolar OLF-2 caused a natriuresis of slower onset reaching its maximum after 60 min and lasting for more than 180 min. This was confirmed also by injection of the active fractions obtained by quantitative TLC.

### Natriuretic factor unrelated to OLF

Finally, I should mention that we described previously a natriuretic compound, which we suggested to be a peptide. Thus, when the pooled post-salt natriuretic urine fraction obtained by gel chromatography (see above) was subjected to repetitive RP-HPLC, a late eluting fraction showed strong natriuretic activity in the bioassay and was associated with a fluorescence peak when treated with o-phthaldialdehyde as a marker for primary amines (11). Amino acid analysis before and after total acid hydrolysis suggested a peptide tentatively containing the amino acids (AA) Asp, Glu, Gly, Phe, and Ser (1, 11). The natriuretic activity was lost after incubation with chymotrypsin, which splits bonds with aromatic AA (2). We found, in addition, that several synthetic (mono-) peptides of di- and tri-AA are significantly natriuretic when injected i.v. (unpublished data).

## Xanthurenic Acid 8-O-β-D-Glucoside and Xanthurenic Acid 8-O-Sulfate as Endogenous Sodium Transport Inhibitors

Cain et al. (4) followed a protocol very similar to that of Kramer et al. for isolation of the natriuretic activity except that they used the urine of uremic patients as source of the inhibitor and a bioassay in (conscious?) uremic rats. As marker for the active material, Cain et al. used changes of the SCC of the isolated frog skin – as we described in 1977 (8) – for monitoring transepithelial sodium transport inhibitory activity. For monitoring its natriuretic effect, the above mentioned bioassay in uremic rats was used. A direct *in vitro*-assay for inhibition of the Na-K-ATPase enzyme by the natriuretic factor or “hormone” was not employed. The authors rather speculate that the natriuretic hormone may act via other sodium pumps in the kidney, e.g., the epithelial sodium channel (ENaC) in the distal tubule.

### Xanthurenic acid derivatives: Effects on epithelial sodium transport

Epithelial sodium transport was measured as changes of SCC and PD in the isolated frog skin. For isolation and identification of the transport inhibitor, one gel chromatographic step and three consecutive HPLC steps were applied. Final identification was achieved by mass (Ms)-, IR-, UV-, and NMR-spectroscopy. Purification of the activity was estimated from UV peak with a characteristic spectrum at 338 nm. Xanthurenic acid 8-O-β-d-glucoside (M_r_ 368) and xanthurenic acid 8-O-sulfate (M_r_ 284) were identified as the endogenous sodium transport (ENaC, Na-K-ATPase) inhibitors.

### Xanthurenic acid derivatives: Natriuretic effects

The material (M_r_ 368) obtained from two HPLC runs was tested for natriuretic effect in their uremic rat bioassay. Urinary sodium excretion rose immediately and reached its maximum approximately 40 min after intra-arterial infusion (5). Urinary volume increased slightly and then decreased to below baseline, i.e., a decrease in urine volume with a rise in urinary osmolality.

A pathophysiological role of xanthurenic acid, a tryptophane derivative, is difficult to envisage as this uremic toxin may inhibit transmembranous sodium transport independent of a potential role as specific circulating natriuretic or sodium transport inhibiting “hormone.” Thus, although Bricker et al. showed that the natriuretic action of the isolated inhibitor paralleled the changes in renal function, as an alternative explanation, it may be reasonable to assume that with the progressive decrease in renal function and the accumulation of toxic metabolites, the rise in fractional sodium excretion may parallel the urinary concentration of the xanthurenic derivatives.

## Summary

Although there is no doubt that an as yet unidentified natriuretic compound can be isolated from human urine by gel filtration and RP-HPLC, whose activity changes in parallel with salt (sodium chloride) intake, i.e., it correlates with salt-balance (low or high salt intake). Therefore, the activity may be related to an as yet unidentified “natriuretic hormone” that is assumed to play a crucial role in the fine-tuning of renal tubular sodium handling and may thus be involved in the long-term body fluid and blood pressure regulation.

We found two Na-K-ATPase inhibitors, the hydrophilic OLF-1 and the lipophilic OLF-2 (1). The hydrophilic form was more potent than the lipophilic one. The lipophilic compound was moderately natriuretic but strong ATPase inhibitor. Both compounds showed UV fluorescence/absorbance of lower intensity in the hydrophilic (hydrated) form. Both enzyme inhibitors showed UV-absorbance, which requires the presence of a dicarboxylic acid-like arrangement (1). In addition, from our data the unknown compound(s) most likely fulfill(s) the criteria for *lactones*.

Unfortunately, for none of the three classes of endogenous sodium transport inhibitors, a physiologic or pathophysiologic role was demonstrated, i.e., no correlation to body fluid and sodium balance or blood pressure was documented. Thus, further studies are required to confirm the structures of the various endogenous factors; final identification of their physiological and pathophysiological significance must await urgent results of additional well-designed studies.

## Conflict of Interest Statement

The author declares that the research was conducted in the absence of any commercial or financial relationships that could be construed as a potential conflict of interest.
